# Electrocardiographic Changes in Pregnant Patients With Congenital Heart Disease

**DOI:** 10.1111/anec.70037

**Published:** 2025-01-06

**Authors:** Maria A. Pabon, Amrit Misra, Kimberlee Gauvreau, Madeline E. Duncan, Ava Conklin, Katherine E. Economy, Fred M. Wu, Thomas Tadros, Anne Marie Valente, For the STORCC Investigators, Shivani R. Aggarwal, Nael Aldweib, Alex Alexander, Laith Alshawabkeh, Tooba Z. Anwer, Nancy Barker, Fernando Baraona Reyes, Yonatan Buber, Jean Marie Carabuena, Matthew Carazo, Christopher DeZorzi, Vedang Diwanji, Sheila Drakeley, Valeria Duarte, Sarah Rae Easter, Gabriele Egidy Assenza, Kimberlee Gauvreau, Julia Graf, Michelle Gurvitz, Daniel Halpern, Amy Harmon, Kelsey Hickey, Jenna Hynes, Caitlyn Joyce, William P. Knapp, Navya Krishna, Michael Landzberg, Matthew Lippmann, Anais Marenco, Sarah E. Miller, Mary Mullen, Alexander Opotowsky, Sara Partington, Tony Pastor, Saraubh Rajpal, Anna Ray, Carla P. Rodriguez‐Monserrate, Carrie Rouse, Zoe Schefter, Keri Shafer, Michael N. Singh, Ada C. Stefanescu Schmidt, Bushra Taha, Allison L. Tsao, Shailendra Upadhyay, Christopher Valle, Sara Villegas‐Keech, Alexandria F. Williams, Fred Wu

**Affiliations:** ^1^ Division of Cardiology, Department of Medicine Brigham and Women's Hospital Boston Massachusetts USA; ^2^ Department of Cardiology Boston Children's Hospital Boston Massachusetts USA; ^3^ Department of Pediatrics Harvard Medical School Boston Massachusetts USA; ^4^ Division of Maternal Fetal Medicine, Department of Obstetrics and Gynecology Brigham and Women's Hospital Boston Massachusetts USA

**Keywords:** adult congenital heart disease, congenital heart disease, electrocardiogram, heart rate, pregnancy

## Abstract

**Background:**

Electrocardiograms (EKGs) are routinely performed in pregnant patients with pre‐existing cardiovascular disease. However, in pregnant patients with congenital heart disease (CHD), EKG changes during gestation have not been explored.

**Methods:**

We performed a retrospective study of pregnant patients with CHD enrolled in the STORCC initiative. Patients were included if they had at least two EKGs across the perinatal period and were grouped by specific conditions: atrial septal defect (ASD), tetralogy of Fallot, congenital pulmonary stenosis, coarctation of the aorta (CoA), bicuspid aortic valve (BAV), systemic right ventricle (SRV), and Fontan circulation. EKG parameters were measured in all available EKGs by two investigators, blinded to diagnosis and time of gestation.

**Results:**

One hundred and seventy pregnant patients were included. There was a statistically significant increase in HR from pre‐pregnancy to third trimester in all groups except for those with Fontan and SRV. Patients with ASD and BAV had a statistically significant increase in their QTc (ASD:13 ms, *p* = 0.017; BAV:7 ms, *p* = 0.018) during pregnancy. QRS duration was shorter (4 ms) in the third trimester for patients with ASD (*p* = 0.033) and CoA (*p* = 0.014). Despite these individual findings, EKG parameters remained within normal limits and regressed to baseline in the postpartum period.

**Conclusions:**

Patients with CHD have statistically significant EKG changes throughout pregnancy, but the values remain within normal limits. Like patients without heart disease, those with CHD increase their HR during pregnancy, except individuals with SRV and Fontan, who appear to lack capacity for physiologic HR augmentation.

## Graphical abstract

Electrocardiographic changes in pregnant patients with congenital heart disease (CHD) participants of the Standardized Outcomes in Reproductive Cardiovascular Care (STORCC) cohort. Patients with atrial septal defect (ASD), Tetralogy of Fallot (TOF), pulmonary stenosis (PS), coarctation of the aorta (CoA), bicuspid aortic valve (BAV), systemic right ventricle (RV), and Fontan circulation were included (Reproduced with permission from OPENPediatrics, Geggel's Congenital Heart Disease Library, edited by Robert Geggel, MD, Boston Children's Hospital).

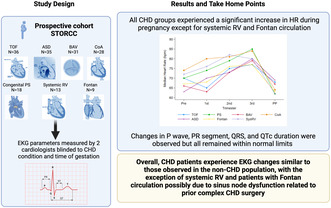

**Abbreviations**
ASD: Atrial septal defect
BAV: Bicuspid aortic valve
CHD: Congenital heart disease
CoA: Coarctation of the aorta
EKG: Electrocardiogram
HR: Heart rate
PS: Pulmonary stenosis
SRV: Systemic right ventricle
STORCC: Standardized Outcomes in Reproductive Cardiovascular Care
TGA: Transposition of the great arteries
TOF: Tetralogy of Fallot

## 1 Introduction

Pregnancy is associated with a range of hemodynamic changes secondary to hormonal and autonomic system adaptations (Jarvis et al. 2012; Sanghavi and Rutherford 2014). During pregnancy, electrocardiograms (EKGs) can serve as a means of surveillance for acute cardiovascular changes (Valente et al. 2019). In individuals with structurally normal hearts, expected EKG changes during pregnancy include sinus tachycardia, shift of the ventricular axis leftward, non‐specific T wave abnormalities, decrease in PR interval, decrease in RR interval, increased P wave duration, and a mild increase in the QTc interval; these intervals remain within normal limits despite these changes (Sanghavi and Rutherford 2014; Angeli, Angeli, and Verdecchia 2014). These changes tend to resolve postpartum, with a return to the pre‐pregnancy baseline values (Jarvis et al. 2012). However, in individuals with congenital heart disease (CHD), EKGs may be abnormal at baseline, depending on the underlying CHD condition, for example, right bundle branch block in those with atrial septal defect (ASD) and tetralogy of Fallot (TOF).

Prior studies have demonstrated that pregnant patients with repaired CHD have an increased incidence of tachyarrhythmia during pregnancy, most commonly non‐sustained ventricular tachycardia and supraventricular tachycardia (Niwa et al. 2007). Recent data suggest that pregnancy may increase the risk of future ventricular arrhythmias in specific CHD conditions, such as TOF, compared to nulliparous patients (Quattrone et al. 2021).

To date, no studies have investigated the longitudinal EKG changes during pregnancy in patients with CHD. Therefore, we sought to investigate EKG changes during pregnancy for patients with CHD and to determine if any significant changes occur in specific CHD conditions.

## 2 Methods

### 2.1 Study Design

The Mass General Brigham Institutional Review Board approved the protocol. All participants provided written informed consent. This is a single‐center analysis of EKGs performed in the pre‐pregnancy, antepartum, and postpartum periods in patients with CHD enrolled in the prospective cohort Standardized Outcomes in Reproductive Cardiovascular Care (STORCC) from November 1, 2011 to December 31, 2022 (Valente et al. 2019). The methodology and inclusion criteria for the STORCC initiative have been previously described by our group (Valente et al. 2019). The cohort was grouped by specific CHD conditions: ASD, TOF, congenital pulmonary stenosis (PS), coarctation of the aorta (CoA), bicuspid aortic valve (BAV), systemic right ventricle (SRV), and Fontan circulation. The SRV group comprised patients with D‐loop transposition of the great arteries (TGA) who had undergone the atrial switch procedure and patients with congenitally corrected, L‐loop TGA. Patients were included who had at least two EKGs during the study period. Specific time intervals were defined as: pre‐pregnancy (within 12 months of conception), first trimester (conception to 13 + 6 weeks gestation), second trimester (14–27 + 6 weeks gestation), third trimester (28 weeks gestation to delivery) and postpartum (up to 3 months following delivery). Exclusion criteria included patients who experienced a spontaneous abortion or ectopic pregnancy or who had fewer than two EKGs during the study period. In patients with more than one eligible pregnancy, only the first pregnancy with at least two available EKGs was included.

Baseline demographics, cardiac history, and comorbid conditions were collected, as well as specific cardiac and obstetric data during the antepartum, intrapartum, and postpartum periods up to 1 year following delivery. Two investigators, blinded to congenital diagnosis and time of gestation, measured the following EKG parameters: P wave duration, PR segment, QRS duration, QT interval, QT corrected (QTc), RR interval, and heart rate (HR). Given that bundle branch blocks and nonspecific intraventricular conduction delays result in a prolonged QRS duration, which can artificially lengthen the QT interval, we applied a cut‐off of ≥ 120 ms for the QRS duration to differentiate normal and prolonged depolarization times to calculate the QT interval (Crow, Hannan, and Folsom 2003). The QTc was calculated using the Bazzett formula, consisting of the QT interval divided by the square root of the RR interval (Crow, Hannan, and Folsom 2003).

### 2.2 Statistical Analysis

Categorical variables are summarized as numbers and percentages, and continuous variables as medians with 25th and 75th percentiles. Within each diagnostic group, electrocardiographic measures were summarized at all five time points, and formal comparisons from pre‐pregnancy to the third trimester were made using the Wilcoxon signed‐rank test. Heart rate was also compared from pre‐pregnancy to postpartum. Within each diagnosis group, change in QTc from pre‐pregnancy to the third trimester was stratified by baseline QTc in the upper quartile of values (highest 25%) versus the lower three quartiles; within each of these two groups, changes were assessed using the signed‐rank test. A two‐sided *p*‐value of < 0.05 was considered statistically significant. Statistical analyses were performed using STATA 16.1 (College Station, TX, USA).

## 3 Results

### 3.1 Baseline Characteristics

Table 1 delineates the baseline demographics of the 170 patients. The most common CHD diagnoses were TOF (21%), ASD (21%), and BAV (18%). Most patients (75% overall) had undergone repair prior to pregnancy, except for the BAV group, in which only 35% had prior intervention. Baseline cardiac medication use was uncommon, with beta blockers being the most common across most CHD types (16% overall). ACE inhibitors and diuretics were the next most common, each used by 4% of patients at baseline. The cohort's median gestational age at delivery was 39 weeks. Patients with Fontan physiology delivered earlier than those with other CHD types, at a median gestational age of 36 [IQR 31, 37] weeks. In total, 67% of patients in the Fontan group delivered preterm (less than 37 weeks gestation) compared to fewer than 25% of patients in each of the other groups. Only one patient (3%) with ASD delivered at less than 37 weeks.

**TABLE 1:** Baseline characteristics by congenital heart disease diagnosis.

	ASD	Tetralogy of Fallot	Pulmonary stenosis	Coarctation of aorta	Bicuspid AV	Systemic RV	Fontan
	*n* = 35	*n* = 36	*n* = 18	*n* = 28	*n* = 31	*n* = 13	*n* = 9
*Demographic information*							
Maternal age at delivery (years)	33 [30, 35]	34 [29, 35]	31 [28, 33]	31 [28, 34]	33 [31, 37]	33 [30, 34]	29 [26, 31]
Gestational age at delivery (wk)	39 [39, 40]	39 [37, 39]	39 [38, 39]	39 [37, 40]	39 [38, 39]	38 [37, 39]	36 [31, 37]
Gestational age at delivery < 37 weeks	1 (3%)	8 (22%)	2 (11%)	3 (11%)	5 (16%)	3 (23%)	6 (67%)
Body mass index	24.0 [22.2, 27.2]	23.5 [20.9, 27.5]	23.9 [22.3, 27.1]	24.8 [21.2, 28.3]	25.7 [22.7, 31.0]	26.1 [24.3, 30.8]	24.7 [24.4, 25.7]
Body mass index ≥ 25	13 (37%)	13 (36%)	7 (39%)	14 (50%)	17 (55%)	8 (62%)	4 (44%)
Race							
White	29 (83%)	26 (72%)	10 (56%)	26 (93%)	29 (94%)	13 (100%)	7 (78%)
Black	5 (14%)	5 (14%)	0 (0%)	1 (4%)	0 (0%)	0 (0%)	1 (11%)
Asian	0 (0%)	5 (14%)	3 (17%)	0 (0%)	0 (0%)	0 (0%)	0 (0%)
Other	1 (3%)	0 (0%)	5 (28%)	1 (4%)	2 (6%)	0 (0%)	1 (11%)
Systolic blood pressure (mmHg)	113 [106, 120]	113 [107, 118]	117 [106, 126]	122 [113, 130]	114 [107, 126]	120 [110, 124]	116 [114, 122]
Diastolic blood pressure (mmHg)	68 [62, 75]	67 [60, 71]	70 [60, 76]	73 [66, 78]	70 [61, 74]	67 [60, 72]	70 [69, 76]
*Medical history*							
CHD							
Repaired	26 (74%)	36 (100%)	12 (67%)	26 (93%)	11 (35%)	8 (62%)	9 (100%)
Unrepaired	9 (26%)	0 (0%)	6 (33%)	2 (7%)	20 (65%)	5 (38%)	0 (0%)
Arrhythmia	2 (6%)	1 (3%)	0 (0%)	1 (4%)	0 (0%)	0 (0%)	0 (0%)
Reduced ejection fraction	0 (0%)	1 (3%)	0 (0%)	0 (0%)	0 (0%)	1 (8%)	1 (11%)
Hypertension	2 (6%)	5 (14%)	2 (11%)	10 (36%)	6 (19%)	1 (8%)	1 (11%)
Diabetes	0 (0%)	1 (3%)	1 (6%)	0 (0%)	1 (3%)	0 (0%)	0 (0%)
Baseline cardiac medications							
ACE inhibitor	0 (0%)	0 (0%)	0 (0%)	0 (0%)	2 (6%)	2 (15%)	3 (33%)
Angiotensin II receptor blocker	0 (0%)	1 (3%)	0 (0%)	0 (0%)	1 (3%)	0 (0%)	0 (0%)
Beta blocker	3 (9%)	8 (22%)	0 (0%)	7 (25%)	5 (16%)	3 (23%)	2 (22%)
Anti‐arrhythmic	0 (0%)	0 (0%)	0 (0%)	0 (0%)	0 (0%)	1 (8%)	0 (0%)
Calcium channel blocker	0 (0%)	0 (0%)	0 (0%)	1 (4%)	1 (3%)	0 (0%)	1 (11%)
Anti‐coagulant	0 (0%)	0 (0%)	0 (0%)	0 (0%)	2 (6%)	0 (0%)	2 (22%)
Diuretic	0 (0%)	1 (3%)	0 (0%)	1 (4%)	1 (3%)	2 (15%)	2 (22%)

*Note:* Values shown are number (percentage) or median [25th, 75th percentiles]. *p* values not included as due to small patient numbers in the systemic RV and Fontan patient groups, the analysis would be underpowered.

Abbreviations: ACE, angiotensin‐converting enzyme; ASD, atrial septal defect; AV, aortic valve; CHD, congenital heart disease; RV, right ventricle; wk, week.

Median baseline body mass index ranged from 23.5 [IQR 20.9, 27.5] kg/m^2^ in the TOF group to 25.7 [IQR 22.7, 31.0] kg/m^2^ in the BAV group. Overall, 45% of patients were overweight or obese prior to becoming pregnant. The next most common comorbidity was systemic hypertension (16% overall), particularly in patients with CoA (36%) or BAV (19%). Less than 2% of patients had a prior history of arrhythmia, reduced ejection fraction, or diabetes mellitus.

Obstetric and neonatal outcomes are summarized in Table 2. Vaginal delivery was achieved in 65% of patients overall; however, cesarean birth occurred more commonly in the subsets of patients with Fontan physiology (66%) and BAV (58%). Unplanned cesarean delivery occurred in 33% of patients with Fontan physiology, 26% of patients with BAV, and 25% of patients with TOF. Neonatal outcomes were generally favorable, with median Apgar scores of 8–9 across the cohort. Infants born to mothers with Fontan surgery tended to have low birth weight; however, this was not statistically significant when adjusting by gestational age.

**TABLE 2:** Obstetric and neonatal characteristics by congenital heart disease diagnosis.

	ASD	Tetralogy of Fallot	Pulmonary stenosis	Coarctation of aorta	Bicuspid AV	Systemic RV	Fontan
	*n* = 35	*n* = 36	*n* = 18	*n* = 28	*n* = 31	*n* = 13	*n* = 9
*Pregnancy*							
New arrhythmia during pregnancy	1 (3%)	0 (0%)	0 (0%)	0 (0%)	0 (0%)	1 (8%)	2 (22%)
*Delivery data*							
Type of delivery							
Vaginal	27 (77%)	24 (67%)	14 (78%)	20 (71%)	13 (42%)	9 (69%)	3 (33%)
Planned cesarean	2 (6%)	3 (8%)	2 (11%)	3 (11%)	10 (32%)	2 (15%)	3 (33%)
Unplanned cesarean	6 (17%)	9 (25%)	2 (11%)	5 (18%)	8 (26%)	2 (15%)	3 (33%)
Twin birth	0 (0%)	0 (0%)	2 (11%)	2 (7%)	1 (3%)	0 (0%)	0 (0%)
Apgar score at 5 min	9 [9, 9] *n* = 35	9 [9, 9] *n* = 36	9 [9, 9] *n* = 20	9 [8, 9] *n* = 29	9 [8, 9] *n* = 31	9 [9, 9] *n* = 12	8 [8, 9] *n* = 9
Birth weight (g)	3310 [3018, 3578] *n* = 35	2903 [2653, 3118] *n* = 36	2899 [2755, 3520] *n* = 20	3125 [2610, 3485] *n* = 29	3175 [2926, 3511] *n* = 31	3075 [2500, 3400] *n* = 13	2175 [1710, 2220] *n* = 9

*Note:* Values shown are number (percentage) or median [25th, 75th percentiles]. *p* values not included as due to small patient numbers in the systemic RV and Fontan patient groups, the analysis would be underpowered.

Abbreviations: ASD, atrial septal defect; AV, aortic valve; g, grams; RV, right ventricle.

### 3.2 EKG Changes During Pregnancy

Table 3 summarizes the availability of EKG data by CHD type. In summary, of the 170 patients, eight had a pre‐pregnancy EKG but no third‐trimester EKG, 51 had a third‐trimester EKG but no pre‐pregnancy EKG, and three had only second and third‐trimester EKGs. Only the 108 patients with both pre‐pregnancy and third‐trimester EKGs were included in the analysis of EKG changes from pre‐pregnancy to the third trimester.

**TABLE 3:** EKG information by congenital heart disease diagnosis.

	ASD	Tetralogy of Fallot	Pulmonary stenosis	Coarctation of aorta	Bicuspid AV	Systemic RV	Fontan
	*n* = 35	*n* = 36	*n* = 18	*n* = 28	*n* = 31	*n* = 13	*n* = 9
*EKG available*							
Pre‐pregnancy EKG	20 (57%)	27 (75%)	8 (44%)	21 (75%)	21 (68%)	13 (100%)	6 (67%)
First trimester EKG	13 (37%)	21 (58%)	6 (33%)	19 (68%)	9 (29%)	9 (69%)	4 (44%)
Second trimester EKG	29 (83%)	31 (86%)	15 (83%)	25 (89%)	23 (74%)	13 (100%)	7 (78%)
Third trimester EKG	34 (97%)	35 (97%)	16 (89%)	25 (89%)	27 (87%)	13 (100%)	9 (100%)
Post‐partum EKG	30 (86%)	31 (86%)	12 (67%)	25 (89%)	27 (87%)	13 (100%)	7 (78%)
Both pre‐pregnancy and third trimester EKG	20 (57%)	26 (72%)	7 (39%)	19 (68%)	17 (55%)	13 (100%)	6 (67%)
Total number of EKGs	4 [3, 4] (2, 5)	4 [3, 5] (2, 5)	3 [2, 4] (2, 5)	4 [3.5, 5] (2, 5)	3 [3, 4] (2, 5)	5 [4, 5] (4, 5)	4 [3, 4] (2, 5)

*Note:* Values shown are number (percent) or median [25th, 75th percentiles] (range).

Abbreviations: ASD, atrial septal defect; AV, aortic valve; EKG, electrocardiogram; RV, right ventricle.

#### 3.2.1 Heart Rate

**FIGURE 1:**
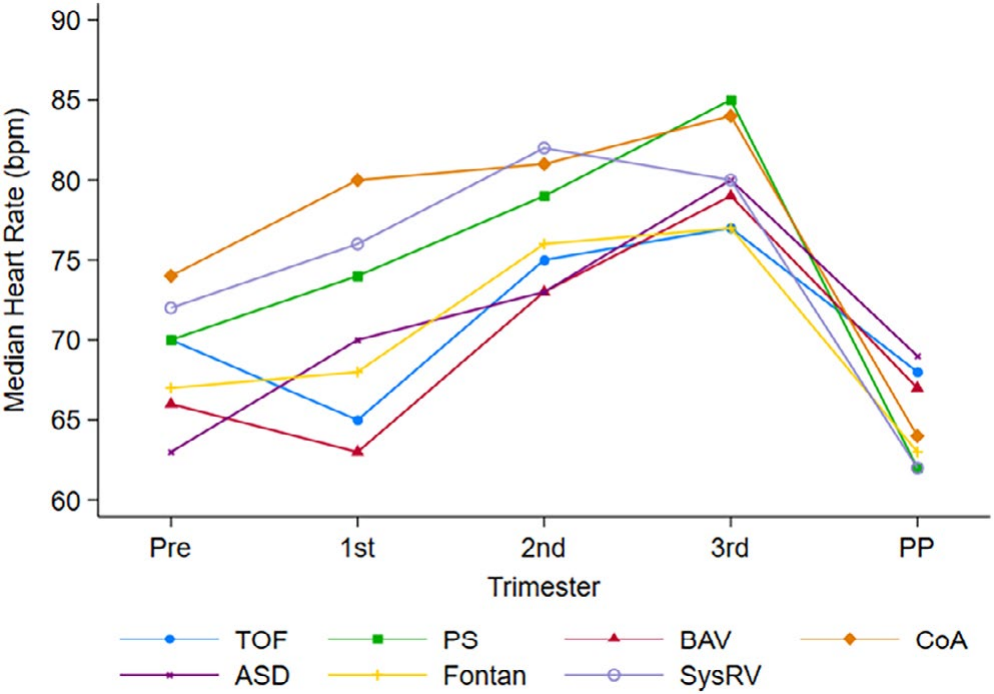
Longitudinal changes in HR in congenital heart disease patients. Abbreviations: ASD, atrial septal defect; BAV, bicuspid aortic valve; CoA, coarctation of the aorta; PS, pulmonary stenosis; SysRV, systemic right ventricle; TOF, tetralogy of Fallot; Pre, pre‐pregnancy; PP, postpartum.

**FIGURE 2:**
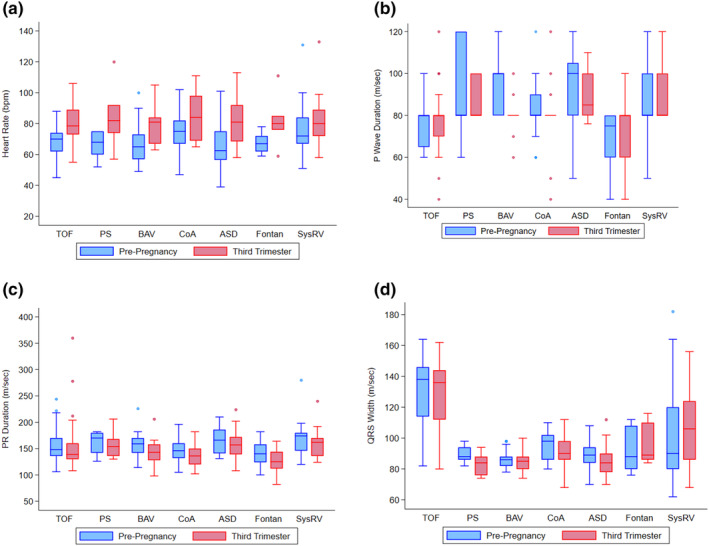
Change in heart rate, P wave, PR interval, and QRS from pre‐pregnancy to third trimester in congenital heart disease patients. Abbreviations: ASD, atrial septal defect; BAV, bicuspid aortic valve; bpm, beats per minute; CoA, coarctation of the aorta; PS, pulmonary stenosis; SysRV, systemic right ventricle; TOF, tetralogy of Fallot. (a) Changes in heart rate. (b) Changes in P wave duration. (c) Changes in PR duration. (d) Changes in QRS width.

There was a statistically significant increase in HR from pre‐pregnancy to the third trimester in all groups except for those with SRV and Fontan (*p* = 0.16 and 0.07, respectively). The median difference in pre‐pregnancy HR to third trimester was eight beats per minute (bpm) in patients with TOF (*p* < 0.001) and PS (*p* = 0.031), 10 bpm in patients with BAV (*p* < 0.001), 14 bpm in patients with ASD (*p* < 0.001) and 16 bpm in patients with CoA (*p* = 0.006). All groups returned to a median HR similar to pre‐pregnancy HR during the post‐partum period, except for those with CoA, who had a median difference between pre‐pregnancy and postpartum HR of −9 bpm (*p* = 0.008). (Figures 1 and 2, and Table S1).

#### 3.2.2 P Wave, PR Segment, and QRS Duration

From pre‐pregnancy to the third trimester, patients with BAV had a significant shortening in their P wave duration (median difference − 10 ms, *p* < 0.001) and PR segment duration (−14 ms, *p* < 0.001). Patients with ASD and CoA also had a significantly shorter PR segment in the third trimester than pre‐pregnancy (−11 ms [*p* = 0.014] and − 6 ms, [*p* = 0.019], respectively), but no significant change in P wave duration. There was no difference in P wave or PR segment duration from pre‐pregnancy to third trimester in the remainder of the groups. Overall, there was no difference between pre‐pregnancy and third‐trimester QRS duration, with the exception of patients with ASD and CoA who exhibited a 4‐ms decrease in QRS duration during the third trimester (*p* = 0.033 and *p* = 0.014, respectively) (Figure 2).

#### 3.2.3 QT and QTc

**FIGURE 3:**
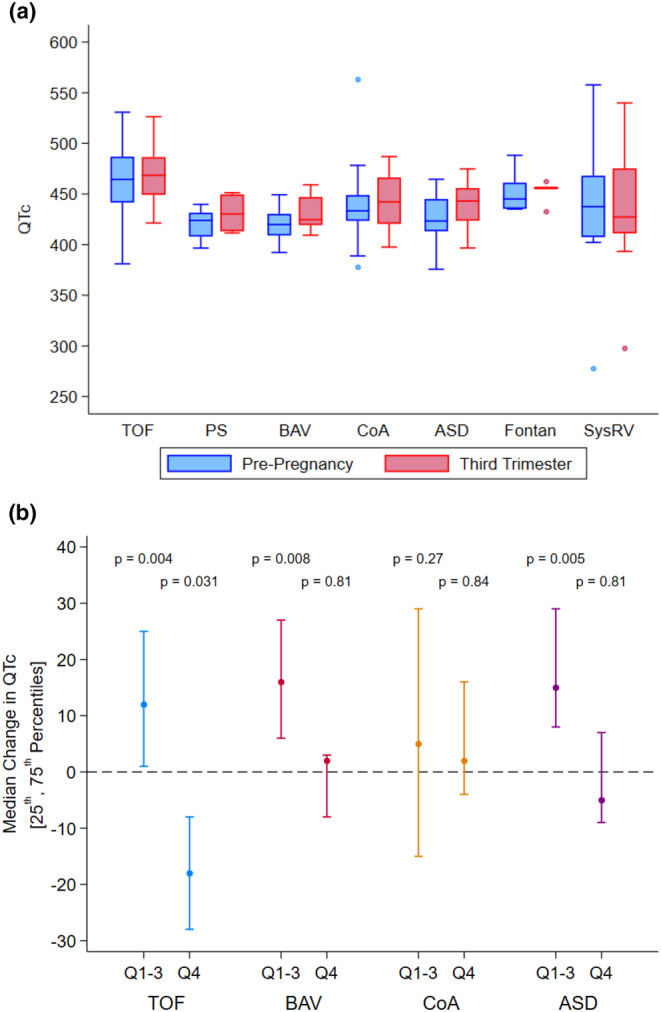
(a) Change in QTc from pre‐pregnancy to third trimester in congenital heart disease patients. (b) Median QTc change in women with baseline QTc in the lower three quartiles (Q1–3) and highest quartile (Q4) by disease type. Abbreviations: ASD, atrial septal defect; BAV, bicuspid aortic valve; CoA, coarctation of the aorta; PS, pulmonary stenosis; SysRV, systemic right ventricle; TOF, tetralogy of Fallot.

There was no statistically significant change in QT or QTc during pregnancy and postpartum for most patient groups except those with ASD and BAV, who had a statistically significant increase in QTc of 13 ms (*p* = 0.017) and 7 ms (*p* = 0.018), respectively (Figure 3). To assess the variation in QTc from baseline, we compared women whose baseline QTc was within the highest quartile to those with lower baseline QTc values. For this, we focused on groups with ASD, TOF, BAV, and CoA, as these conditions had a sufficiently large sample size for robust analysis. Except for patients with CoA, a significant prolongation of QTc during the third trimester of pregnancy was predominantly observed in women whose baseline QTc was in the lower three quartiles. Conversely, patients whose baseline QTc resided in the highest quartile did not experience a statistically significant change in QTc duration throughout their pregnancy (Figure 3b).

## 4 Discussion

This study documents longitudinal EKG changes during pregnancy for patients with various types of CHD. Important and novel findings include: (1) Patients with SRV and Fontan do not experience a significant increase in HR in the third trimester of pregnancy, as is seen in patients with other CHD subgroups and in patients without CHD; (2) some subgroups of CHD experience statistically significant decreases in P wave duration, PR, and QRS through the third trimester of pregnancy, similar to patients without CHD, but these intervals remain within normal limits, and there was no evidence of increased interval durations to suggest atrioventricular or intraventricular conduction disturbances; (3) the ASD and BAV subgroups had statistically significant increases in QTc in pregnancy and postpartum; nonetheless, the increase was not clinically relevant, with a less than 5% increase over the baseline QTc, and with no significant change in QTc in patients whose baseline QTc was in the highest quartile.

While electrocardiographic changes during pregnancy in patients with structurally normal hearts have been studied, there are very limited data regarding these changes in pregnant patients with CHD. Historical obstetric literature suggests that HR typically rises by 10–20 bpm in pregnant patients, peaking during the third trimester (Blackburn 2013; Carruth et al. 1981; Clapp and Capeless 1997; Mahendru et al. 2014; Loerup et al. 2019). However, this concept is mostly derived from studies limited by small sample sizes and considerable disease heterogeneity. Establishing a consensus on what constitutes a physiological rise in HR during pregnancy is critical to improving early detection of clinical deterioration in pregnant patients with and without cardiovascular disease.

Interestingly, in our small subset of patients with SRV and Fontan physiology, there was not a statistically significant increase in HR across gestation. There are several possible explanations for this finding. Sinus node dysfunction is known to occur in up to 60% of patients with Fontan physiology and 48% of patients with D‐TGA after atrial switch (Cohen et al. 1998; Balaji et al. 2014; Dilawar et al. 2003; Dos et al. 2005). These surgeries may limit the ability to increase HR during pregnancy due to damage to the sinus node or its artery and to post‐surgical adverse atrial remodeling (Stephenson et al. 2010). Additionally, 14.7% of our total cohort were on medications that may limit HR response or slow conduction, including 23% and 22% of the SRV and Fontan patients, respectively, which may have contributed to lack of HR augmentation across trimesters.

Regarding specific EKG intervals, statistically significant changes did occur, but importantly, none were outside of the range of normal, and these values returned to baseline levels following delivery. These findings are similar to those in patients without CHD, who also have small changes in EKG intervals during pregnancy without significantly abnormal findings (Jarvis et al. 2012; Sanghavi and Rutherford 2014; Angeli, Angeli, and Verdecchia 2014). Specifically, shortening of the P wave duration, QRS duration, and PR interval have been reported in normal pregnancies (Carruth et al. 1981; Ananthakrishnan et al. 2020; Sumalatha, Jyotsna, and Indrani 2017). In our cohort, we observed similar EKG changes in ASD, BAV, and CoA groups, presumably related to the more pronounced increase in HR relative to the other subgroups.

The QT interval represents ventricular depolarization and repolarization. Prior studies have suggested that the QT interval tends to shorten in healthy pregnancies (Carruth et al. 1981; Baumert et al. 2010; Carpenter et al. 2015), whereas the QTc may remain unchanged or prolong slightly during the third trimester (Carruth et al. 1981; Ananthakrishnan et al. 2020; Tanindi et al. 2016), possibly reflecting the relationship of the QT with HR (Baumert et al. 2010). QTc changes during pregnancy have been attributed to changes in neurohormonal regulation of the myocardium and the physical rearrangement of the abdominal and thoracic organs during pregnancy, potentially affecting ventricular electrical activity (Lechmanová, Parízek, et al. 2002; Lechmanová, Kittnar, et al. 2002). Indeed, animal studies have revealed important sex‐related differences in cardiac repolarization (Trépanier‐Boulay et al. 2001) which may be linked to the modulatory effects of estrogen (Pham et al. 2001). In our study, individuals with ASD and BAV exhibited a slight but statistically significant prolongation of the QTc which, nonetheless, remained within normal limits. This was not observed in patients with TOF, SRV, and Fontan physiology, potentially due to prior cardiac interventions leading to baseline electrophysiologic changes that prevented a change in these intervals during pregnancy. Further research is warranted to understand the complex interplay of these factors and their impact on cardiac electrophysiology during pregnancy.

Our study has several limitations. First, the sample size is small, particularly in the Fontan and SRV subgroups, which partially restricted our statistical power to detect EKG alterations during pregnancy. Additionally, the absence of a control group limits comparative analysis as serial EKGs are not standard practice for healthy pregnant individuals. Furthermore, the diversity in disease pathophysiology and hemodynamic profiles among our CHD subgroups limits intergroup comparisons. The small numbers in our study also make it underpowered to detect significant changes between CHD groups.

Our study also has several strengths. To our knowledge, this is the first study examining EKG changes in a prospective cohort of pregnant individuals with CHD. The STORCC protocol calls for each participant to undergo EKG during each trimester and in the postpartum period. Additionally, all data collected in STORCC are standardized, including comprehensive clinical characteristics and pregnancy outcomes.

## 5 Conclusions

This study provides insights into the EKG changes experienced by individuals with different subtypes of CHD during pregnancy. Similar to the general population, the majority of individuals with CHD exhibit significant changes in HR and EKG parameters during pregnancy, with most reverting to pre‐pregnancy baseline during the postpartum period. This data can assist providers caring for patients with CHD during pregnancy, providing guidance as to expected physiologic EKG changes and helping them identify abnormal findings when present. Larger studies are needed to confirm our findings and to further elucidate the cardiovascular changes detected by EKG during pregnancy in the CHD population.

## Author Contributions

All authors contributed to the conception and design of the study, or acquisition of data, analysis and interpretation of data, drafting the article or revising it critically for important intellectual content. Drs. Maria A. Pabon and Anne Marie Valente gave final approval of the version to be submitted.

## Ethics Statement

The study was conducted in accordance with the Declaration of Helsinki, and approved by the Institutional Review Board of Mass General Brigham.

## Consent

Informed consent was obtained from all subjects involved in the study.

## Conflicts of Interest

The authors declare no conflicts of interest.

## Supporting information


Table S1.

## Data Availability

The data that support the findings of this study are available on request from the corresponding author. The data are not publicly available due to privacy or ethical restrictions.
