# Supplementary material for: Electrocardiographic Changes in Pregnant Patients With Congenital Heart Disease
Source: Ann Noninvasive Electrocardiol. 2025 Jan 6;30(1):e70037. doi: 10.1111/anec.70037 (PMC11705495; doi:10.1111/anec.70037)
Supplement: Supplementary file 1 — Table S1. [file ANEC-30-e70037-s001.docx]

**Table S1:** **Changes in Electrocardiographic (EKG) Parameters During Pregnancy and Postpartum by Congenital Heart Disease Type**

|  |  | **Tetralogy of Fallot (36 patients)** | | | | | ***Comparison of Pre-Pregnancy to Third Trimester*** | | | ***Comparison of Pre-Pregnancy to Post-Partum*** | | |
| --- | --- | --- | --- | --- | --- | --- | --- | --- | --- | --- | --- | --- |
|  |  | Pre-Pregnancy | First Trimester | Second Trimester | Third Trimester | Post-Partum | Number with Values at Both Time Points | Median Difference Pre to Third Trimester | P Value | Number with Values at Both Time Points | Median Difference Pre to Post-Partum | P Value |
| Heart Rate (bpm) | Median | 70 | 65 | 75 | 77 | 68 | 26 | 8 | <0.001 | 24 | -1 | 0.84 |
|  | 25th, 75th Percentiles | 62, 74 | 60, 79 | 69, 84 | 72, 86 | 60, 74 |  |  |  |  |  |  |
| P Wave Duration (m/sec) | Median | 80 | 80 | 80 | 80 | 80 | 24 | 0 | 0.90 | 22 | 0 | 0.50 |
|  | 25th, 75th Percentiles | 70, 80 | 70, 80 | 70, 80 | 70, 80 | 80, 100 |  |  |  |  |  |  |
| PR Duration (m/sec) | Median | 148 | 156 | 146 | 140 | 158 | 26 | -5 | 0.27 | 24 | 6 | 0.097 |
|  | 25th, 75th Percentiles | 136, 184 | 129, 180 | 130, 162 | 130, 162 | 140, 176 |  |  |  |  |  |  |
| QRS Width (m/sec) | Median | 138 | 134 | 136 | 134 | 140 | 26 | -2 | 0.86 | 24 | 6 | 0.063 |
|  | 25th, 75th Percentiles | 114, 146 | 120, 140 | 124, 144 | 110, 142 | 114, 154 |  |  |  |  |  |  |
| QTc | Median | 445 | 449 | 449 | 449 | 443 | 26 | 8 | 0.22 | 24 | -2 | 0.44 |
|  | 25th, 75th Percentiles | 431, 469 | 435, 468 | 441, 471 | 436, 466 | 426, 458 |  |  |  |  |  |  |

*This table presents the median values and changes of EKG parameters across different stages of pregnancy and postpartum in patients with congenital heart disease. BPM, beats per minute; m/sec, millimeter per second

|  |  | **Pulmonary Stenosis (18 patients)** | | | | | ***Comparison of Pre-Pregnancy to Third Trimester*** | | | ***Comparison of Pre-Pregnancy to Post-Partum*** | | |
| --- | --- | --- | --- | --- | --- | --- | --- | --- | --- | --- | --- | --- |
|  |  | Pre-Pregnancy | First Trimester | Second Trimester | Third Trimester | Post-Partum | Number with Values at Both Time Points | Median Difference Pre to Third Trimester | P Value | Number with Values at Both Time Points | Median Difference Pre to Post-Partum | P Value |
| Heart Rate (bpm) | Median | 70 | 74 | 79 | 85 | 62 | 7 | 8 | 0.031 | 7 | -4 | 0.11 |
|  | 25th, 75th Percentiles | 63, 75 | 65, 77 | 70, 86 | 75, 97 | 51, 70 |  |  |  |  |  |  |
| P Wave Duration (m/sec) | Median | 85 | 100 | 100 | 80 | 80 | 7 | 0 | 1.0 | 7 | 0 | 0.75 |
|  | 25th, 75th Percentiles | 80, 110 | 80, 100 | 80, 100 | 80, 100 | 80, 85 |  |  |  |  |  |  |
| PR Duration (m/sec) | Median | 173 | 165 | 158 | 145 | 165 | 7 | 0 | 0.69 | 7 | -6 | 0.50 |
|  | 25th, 75th Percentiles | 148, 181 | 146, 172 | 144, 174 | 136, 154 | 150, 171 |  |  |  |  |  |  |
| QRS Width (m/sec) | Median | 88 | 90 | 84 | 81 | 94 | 7 | -2 | 0.19 | 7 | 4 | 0.80 |
|  | 25th, 75th Percentiles | 87, 91 | 86, 96 | 80, 92 | 75, 87 | 85, 103 |  |  |  |  |  |  |
| QTc | Median | 423 | 431 | 436 | 437 | 426 | 7 | 11 | 0.11 | 7 | -15 | 0.58 |
|  | 25th, 75th Percentiles | 411, 430 | 421, 447 | 419, 438 | 419, 443 | 412, 438 |  |  |  |  |  |  |

*This table presents the median values and changes of EKG parameters across different stages of pregnancy and postpartum in patients with congenital heart disease. BPM, beats per minute; m/sec, millimeter per second

|  |  | **Bicuspid AV (31 patients)** | | | | | ***Comparison of Pre-Pregnancy to Third Trimester*** | | | ***Comparison of Pre-Pregnancy to Post-Partum*** | | |
| --- | --- | --- | --- | --- | --- | --- | --- | --- | --- | --- | --- | --- |
|  |  | Pre-Pregnancy | First Trimester | Second Trimester | Third Trimester | Post-Partum | Number with Values at Both Time Points | Median Difference Pre to Third Trimester | P Value | Number with Values at Both Time Points | Median Difference Pre to Post-Partum | P Value |
| Heart Rate (bpm) | Median |  | 63 | 73 | 79 | 67 | 18 | 10 | <0.001 | 18 | 1 | 0.69 |
|  | 25th, 75th Percentiles | 57, 73 | 62, 75 | 68, 87 | 67, 86 | 57, 72 |  |  |  |  |  |  |
| P Wave Duration (m/sec) | Median | 100 | 80 | 80 | 80 | 80 | 17 | -10 | <0.001 | 18 | -3 | 0.30 |
|  | 25th, 75th Percentiles | 80, 100 | 80, 90 | 80, 85 | 80, 85 | 80, 100 |  |  |  |  |  |  |
| PR Duration (m/sec) | Median | 162 | 158 | 156 | 143 | 152 | 18 | -14 | <0.001 | 18 | 0 | 0.59 |
|  | 25th, 75th Percentiles | 148, 170 | 144, 172 | 130, 168 | 128, 158 | 142, 170 |  |  |  |  |  |  |
| QRS Width (m/sec) | Median | 86 | 86 | 82 | 83 | 86 | 18 | 0 | 0.57 | 18 | -1 | 0.59 |
|  | 25th, 75th Percentiles | 82, 88 | 82, 90 | 80, 90 | 79, 89 | 84, 92 |  |  |  |  |  |  |
| QTc | Median | 420 | 417 | 431 | 430 | 426 | 18 | 7 | 0.018 | 18 | -1 | 0.55 |
|  | 25th, 75th Percentiles | 411, 427 | 416, 434 | 416, 447 | 421, 446 | 406, 436 |  |  |  |  |  |  |

*This table presents the median values and changes of EKG parameters across different stages of pregnancy and postpartum in patients with congenital heart disease. BPM, beats per minute; m/sec, millimeter per second

|  |  | **Coarctation of Aorta (28 patients)** | | | | | ***Comparison of Pre-Pregnancy to Third Trimester*** | | | ***Comparison of Pre-Pregnancy to Post-Partum*** | | |
| --- | --- | --- | --- | --- | --- | --- | --- | --- | --- | --- | --- | --- |
|  |  | Pre-Pregnancy | First Trimester | Second Trimester | Third Trimester | Post-Partum | Number with Values at Both Time Points | Median Difference Pre to Third Trimester | P Value | Number with Values at Both Time Points | Median Difference Pre to Post-Partum | P Value |
| Heart Rate (bpm) | Median | 74 | 80 | 81 | 84 | 64 | 19 | 16 | 0.006 | 20 | -9 | 0.008 |
|  | 25th, 75th Percentiles | 67, 79 | 73, 90 | 74, 89 | 78, 99 | 58, 71 |  |  |  |  |  |  |
| P Wave Duration (m/sec) | Median | 80 | 80 | 80 | 80 | 80 | 11 | 0 | 0.50 | 10 | 0 | 0.75 |
|  | 25th, 75th Percentiles | 80, 90 | 80, 90 | 80, 90 | 80, 80 | 80, 80 |  |  |  |  |  |  |
| PR Duration (m/sec) | Median | 146 | 140 | 132 | 140 | 136 | 19 | -6 | 0.019 | 20 | -5 | 0.18 |
|  | 25th, 75th Percentiles | 134, 160 | 128, 158 | 126, 154 | 124, 150 | 128, 152 |  |  |  |  |  |  |
| QRS Width (m/sec) | Median | 96 | 92 | 86 | 90 | 96 | 19 | -4 | 0.014 | 20 | 0 | 0.23 |
|  | 25th, 75th Percentiles | 86, 102 | 84, 98 | 82, 92 | 82, 94 | 90, 100 |  |  |  |  |  |  |
| QTc | Median | 429 | 441 | 439 | 445 | 431 | 19 | 5 | 0.33 | 20 | -1 | 0.70 |
|  | 25th, 75th Percentiles | 423, 447 | 427, 451 | 418, 446 | 434, 463 | 409, 450 |  |  |  |  |  |  |

*This table presents the median values and changes of EKG parameters across different stages of pregnancy and postpartum in patients with congenital heart disease. BPM, beats per minute; m/sec, millimeter per second

|  |  | **ASD (35 patients)** | | | | | ***Comparison of Pre-Pregnancy to Third Trimester*** | | | ***Comparison of Pre-Pregnancy to Post-Partum*** | | |
| --- | --- | --- | --- | --- | --- | --- | --- | --- | --- | --- | --- | --- |
|  |  | Pre-Pregnancy | First Trimester | Second Trimester | Third Trimester | Post-Partum | Number with Values at Both Time Points | Median Difference Pre to Third Trimester | P Value | Number with Values at Both Time Points | Median Difference Pre to Post-Partum | P Value |
| Heart Rate (bpm) | Median | 63 | 70 | 73 | 80 | 69 | 20 | 14 | <0.001 | 19 | 5 | 0.45 |
|  | 25th, 75th Percentiles | 57, 75 | 60, 76 | 70, 83 | 69, 93 | 58, 77 |  |  |  |  |  |  |
| P Wave Duration (m/sec) | Median | 100 | 90 | 100 | 90 | 95 | 20 | 0 | 0.43 | 19 | 10 | 0.44 |
|  | 25th, 75th Percentiles | 80, 105 | 80, 110 | 80, 100 | 80, 100 | 80, 100 |  |  |  |  |  |  |
| PR Duration (m/sec) | Median | 166 | 148 | 160 | 156 | 157 | 20 | -11 | 0.014 | 19 | 4 | 0.054 |
|  | 25th, 75th Percentiles | 141, 186 | 134, 168 | 140, 174 | 136, 168 | 142, 184 |  |  |  |  |  |  |
| QRS Width (m/sec) | Median | 89 | 84 | 88 | 84 | 90 | 20 | -4 | 0.033 | 19 | 2 | 0.37 |
|  | 25th, 75th Percentiles | 84, 94 | 80, 91 | 82, 92 | 80, 92 | 84, 98 |  |  |  |  |  |  |
| QTc | Median | 423 | 439 | 444 | 442 | 430 | 20 | 13 | 0.017 | 19 | 8 | 0.045 |
|  | 25th, 75th Percentiles | 413, 445 | 431, 448 | 434, 454 | 428, 454 | 416, 449 |  |  |  |  |  |  |

*This table presents the median values and changes of EKG parameters across different stages of pregnancy and postpartum in patients with congenital heart disease. BPM, beats per minute; m/sec, millimeter per second

|  |  | **Fontan (9 patients)** | | | | | ***Comparison of Pre-Pregnancy to Third Trimester*** | | | ***Comparison of Pre-Pregnancy to Post-Partum*** | | |
| --- | --- | --- | --- | --- | --- | --- | --- | --- | --- | --- | --- | --- |
|  |  | Pre-Pregnancy | First Trimester | Second Trimester | Third Trimester | Post-Partum | Number with Values at Both Time Points | Median Difference Pre to Third Trimester | P Value | Number with Values at Both Time Points | Median Difference Pre to Post-Partum | P Value |
| Heart Rate (bpm) | Median | 67 | 68 | 76 | 77 | 63 | 6 | 11 | 0.16 | 4 | -4 | 0.63 |
|  | 25th, 75th Percentiles | 62, 72 | 67, 71 | 72, 85 | 74, 85 | 60, 92 |  |  |  |  |  |  |
| P Wave Duration (m/sec) | Median | 75 | 65 | 60 | 80 | 80 | 6 | 5 | 0.63 | 4 | 10 | 0.50 |
|  | 25th, 75th Percentiles | 60, 80 | 50, 75 | 40, 80 | 60, 100 | 40, 80 |  |  |  |  |  |  |
| PR Duration (m/sec) | Median | 140 | 120 | 136 | 130 | 144 | 6 | -8 | 0.16 | 4 | 7 | 0.50 |
|  | 25th, 75th Percentiles | 124, 158 | 98, 166 | 100, 154 | 112, 144 | 108, 154 |  |  |  |  |  |  |
| QRS Width (m/sec) | Median | 88 | 96 | 98 | 98 | 100 | 6 | 5 | 0.28 | 4 | 6 | 0.13 |
|  | 25th, 75th Percentiles | 80, 108 | 88, 109 | 84, 110 | 88, 104 | 96, 110 |  |  |  |  |  |  |
| QTc | Median | 445 | 440 | 438 | 456 | 445 | 6 | -1 | 1.0 | 4 | -7 | 0.63 |
|  | 25th, 75th Percentiles | 435, 461 | 429, 468 | 414, 458 | 451, 457 | 439, 468 |  |  |  |  |  |  |

*This table presents the median values and changes of EKG parameters across different stages of pregnancy and postpartum in patients with congenital heart disease. BPM, beats per minute; m/sec, millimeter per second

|  |  | **Systemic RV (13 patients)** | | | | | ***Comparison of Pre-Pregnancy to Third Trimester*** | | | ***Comparison of Pre-Pregnancy to Post-Partum*** | | |
| --- | --- | --- | --- | --- | --- | --- | --- | --- | --- | --- | --- | --- |
|  |  | Pre-Pregnancy | First Trimester | Second Trimester | Third Trimester | Post-Partum | Number with Values at Both Time Points | Median Difference Pre to Third Trimester | P Value | Number with Values at Both Time Points | Median Difference Pre to Post-Partum | P Value |
| Heart Rate (bpm) | Median | 72 | 76 | 82 | 80 | 62 | 13 | 2 | 0.069 | 13 | -4 | 0.14 |
|  | 25th, 75th Percentiles | 67, 84 | 61, 86 | 69, 90 | 72, 89 | 56, 80 |  |  |  |  |  |  |
| P Wave Duration (m/sec) | Median | 80 | 100 | 90 | 80 | 100 | 13 | 0 | 1.0 | 12 | 5 | 0.094 |
|  | 25th, 75th Percentiles | 80, 100 | 80, 100 | 80, 100 | 80, 100 | 90, 100 |  |  |  |  |  |  |
| PR Duration (m/sec) | Median | 174 | 150 | 162 | 162 | 172 | 13 | -12 | 0.15 | 12 | -2 | 0.99 |
|  | 25th, 75th Percentiles | 146, 180 | 132, 174 | 140, 178 | 136, 170 | 148, 177 |  |  |  |  |  |  |
| QRS Width (m/sec) | Median | 90 | 104 | 98 | 106 | 108 | 13 | 4 | 0.21 | 13 | 12 | 0.016 |
|  | 25th, 75th Percentiles | 80, 120 | 88, 124 | 88, 124 | 86, 124 | 82, 132 |  |  |  |  |  |  |
| QTc | Median | 438 | 434 | 430 | 427 | 441 | 13 | -10 | 0.54 | 13 | -8 | 0.31 |
|  | 25th, 75th Percentiles | 408, 464 | 424, 465 | 418, 468 | 411, 457 | 399, 460 |  |  |  |  |  |  |

*This table presents the median values and changes of EKG parameters across different stages of pregnancy and postpartum in patients with congenital heart disease. BPM, beats per minute; m/sec, millimeter per second
